# Urazole-Functionalized Carbon Nanotubes as Artificial DNA Strands and Their *In Vivo* Toxicity

**DOI:** 10.1021/acsnano.5c16279

**Published:** 2026-01-28

**Authors:** Shwu-Chen Tsay, Deepa R. Landge, Wen-Chieh Huang, Uttam Patil, Jia-Cherng Horng, Chun-Cheng Lin, Yu-Chen Hu, Syed N. Barmaver, Oliver I. Wagner, Jih Ru Hwu

**Affiliations:** † Department of Chemistry, 34881 National Tsing Hua University, Hsinchu 300044, Taiwan; ‡ Frontier Research Center on Fundamental and Applied Sciences of Matters, 34881 National Tsing Hua University, Hsinchu 300044, Taiwan; § Department of Chemical Engineering, National Tsing Hua University, Hsinchu 300044, Taiwan; ∥ Institute of Molecular and Cellular Biology, Department of Life Science, National Tsing Hua University, Hsinchu 300044, Taiwan

**Keywords:** DNA groove binder, urazole, functionalized carbon nanotube, hybridization, *in vivo* toxicity, triplex DNA

## Abstract

Single-walled carbon nanotubes (SWCNTs) are grafted with multiple phenoxy–triazole–(ethylene glycol) ligands, whose terminals are uniformly functionalized with DNA-binding moieties. Each binder contains one to three binding sites for imidazolidin-2-one, hydantoin, or urazole. Entwinement of these “artificial strands” with ss- and dsDNAs forms pseudoduplex or pseudotriplex DNA structures, respectively. The hybridization preference between the functionalized SWCNTs (i.e., *f*-SWCNTs **1a**–**c**) and ssDNA is investigated using homo-oligomers ss-dA_25_, ss-dT_25_, and ss-dC_25_. Results show that ss-dA_25_ bound strongly to *f*-SWCNT **1c** bearing urazoles, ss-dT_25_ bound exclusively to **1c**, and ss-dC_25_ bound selectively to **1a** (imidazolidin-2-one) and **1b** (hydantoin). For the formation of pseudotriplets with duplex oligomers dA_25_•dT_25_ and dG_25_•dC_25_ at pH 7.4, d­(A•T) pairs bound strongly to **1c** but weakly to **1a** and **1b**. Conversely, d­(G•C) pairs bound strongly to **1a** and moderately to **1b** and **1c**. Under acidic conditions (pH 5.4), **1c** exhibits the strongest binding, while **1a** and **1b** show moderate affinity. These findings highlight the potent binding capability and nucleobase selectivity of *f*-SWCNT **1c** functionalized with urazoles. Biocompatibility is also assessed—touch responses and thrashing assays are performed to evaluate the ecotoxicity of shortened *f*-SWCNT **1c** on the nervous system of *C. elegans*. No significant toxicity is observed up to concentrations of 250 μg/mL, with *f*-SWCNT **1c** ∼2500-fold less toxic than simple SWCNT–COOH. These results suggest that the newly developed *f*-SWCNT **1c** is not only highly biocompatible but also holds great promise for broad biological applications.

## Graphical abstract

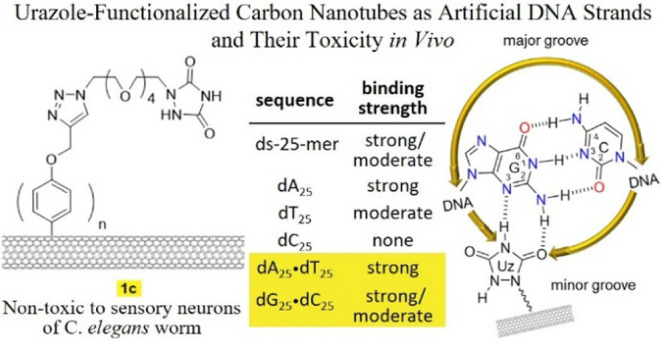

## Introduction

Recently, triplex-DNA formation has been achieved by using functionalized single-walled carbon nanotubes (*f*-SWCNTs) as pseudo third strands capable of hybridizing with dsDNA.^1^ The surfaces of *f*-SWCNTs **1a**–**c** as shown in Chart [Fig chart1] are grafted with multiple amphipathic ligands bearing DNA-binding moieties at the terminal: imidazolidin-2-one (**2a**), imidazolidine-2,4-dione (hydantoin, **2b**), and 1,2,4-triazolidine-3,5-dione (urazole, **2c**). These *N*-heterocyclic DNA binders (*N*-HDBs) contain an adjacent proton donor and proton acceptor to enable hydrogen-bond formation with the amino and carbonyl groups of DNA bases (see Chart [Fig chart1]). Their compact five-membered ring structures also insert into either the minor or major grooves of dsDNA to facilitate Hoogsteen-type base pairing, characteristic of triplex DNA. In addition, entanglement of ssDNA with *f*-SWCNTs **1a**–**c** produces pseudo duplexes through Watson–Crick-type hydrogen bonding, with the carbon nanotubes acting as a complementary strand to DNA. The formation of both pseudo duplex- and triplex-DNA structures is confirmed by circular dichroism (CD) spectroscopy, scanning electron microscopy, high-resolution transmission electron microscopy, and atomic force microscopy.^1^

**1 chart1:**
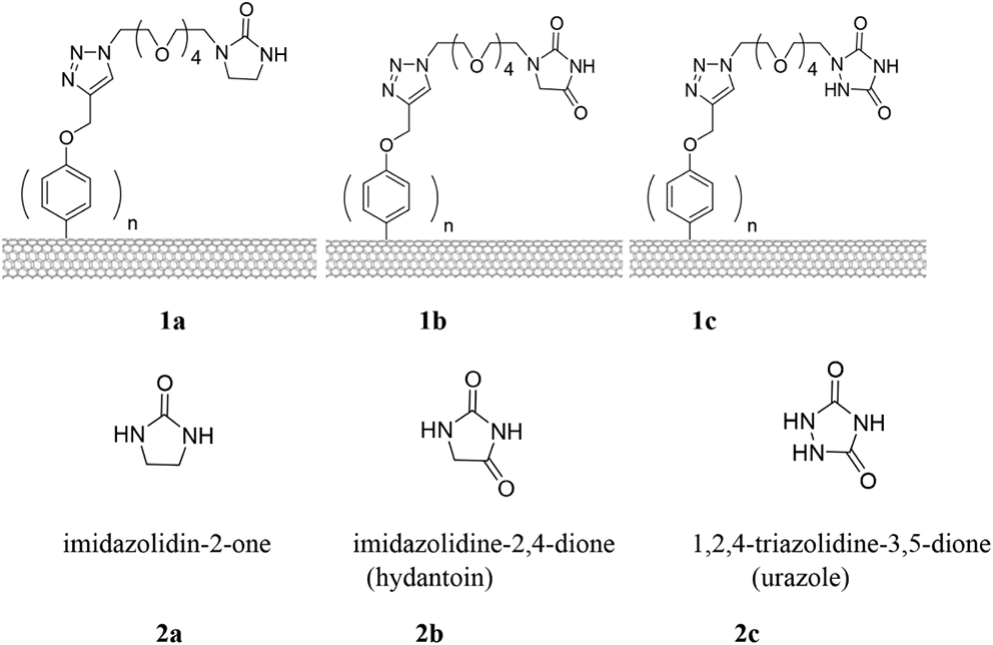
Structures of Functionalized Single-Walled Carbon Nanotubes **1a–c** and *N*-Heterocyclic DNA Binders **2a–c**

Formation of triple-helical DNA structures from a mixture of dsDNA and an oligonucleotide as a third strand typically involves Hoogsteen or reverse Hoogsteen hydrogen bonding.^2^ This process, which is energetically unfavorable, occurs in the major groove of dsDNA at oligopyrimidine•oligopurine sequences. Oligonucleotides longer than the persistence length may undergo folding or exhibit three-dimensional random motion.^3^ These flexible conformational properties stand in sharp contrast with the intrinsic rigidity imposed by the central sp^2^ carbon framework of *f*-SWCNTs **1a**–**c**. Consequently, the hydrogen-bonding interactions between dsDNA and nanotubes **1a**–**c** are expected to differ fundamentally from those formed between dsDNA and an oligonucleotide strand. To compensate for the structural disparity in rigidity, we designed the ligands on the surfaces of *f*-SWCNTs **1a**–**c** with flexible poly­(ethylene glycol) (PEG) spacers. These spacers may act as molecular “tentacles,” which position the sequential DNA-binding groups at the nanotube termini to hybridize effectively with nucleobases in dsDNA.

To elucidate the bonding patterns in the resultant “pseudo” complementary DNAs (i.e., DNA@*f*-SWCNTs **1a**–**c**), we employed a series of homo-oligomers to hybridize individually with *f*-SWCNTs **1a**–**c**. Single-stranded 25-mer oligonucleotides, including ss-dA_25_, ss-dT_25_, and ss-dC_25_, were used to probe the binding strength of (*N*-HDB)_*n*_•(nucleobase)_*n*_ interactions within the pseudo duplex structures. In these systems, the *N*-heterocyclic DNA binders (*N*-HDBs) were imidazolidin-2-one in **1a**, hydantoin in **1b**, and urazole in **1c,** while the nucleobases were A, T, or C. The ss-dG_25_ was excluded because it readily forms dG_25_-quadruplex via intrastranded interactions.^4,5^ Furthermore, the homoduplex oligomers dA_25_•dT_25_ and dG_25_•dC_25_ were employed to investigate the interactions of (*N*-HDB)_*n*_–(A•T)_*n*_ and (*N*-HDB)_*n*_–(G•C)_*n*_ within pseudo triplex-DNA complexes. This approach enabled us to determine the preferred hydrogen-bonding modes between *f*-SWCNTs **1a**–**c,** acting as artificial DNA strands, and individual nucleobases or base pairs in DNA. Circular dichroism (CD) spectroscopy was then applied to monitor the complex formation and to assess pH-dependent effects on secondary structures, conformational changes, and perturbations in the geometry of complementary nucleic acids.^6^

*f*-SWCNTs have emerged as attractive materials for diverse applications, including drug delivery, nucleic acid analysis, electron emitters, sensors, transistors, nanocomposites, and so forth.^7,8^ Although long SWCNTs exhibit excellent optical properties, many biological applications require shortened nanotubes, ideally in the range of ∼40–400 nm.^9^

Nonfunctionalized SWCNTs are known to be toxic,^10^ largely due to their tendency to aggregate, which leads to tissue accumulation and physiological complications. Covalent or noncovalent functionalization with small molecules or ligands can effectively reduce such aggregation.^11^ Thus, the use of short, functionalized SWCNTs offers the dual benefit of improved dispersibility and reduced toxicity.

Herein, we report our investigated results for hybridization preferences between DNA nucleobases and *f*-SWCNTs **1a**–**c**. The binding strength and interaction modes between these artificial strands and DNA are systematically characterized. Moreover, we demonstrate an approach to obtain an *f*-SWCNT with minimal toxicity and advance its potential for safe and broad biological applications.

## Results and Discussion

### Hybridization of Single-Stranded Homo-Oligomers with *f*-SWCNTs **1a–c**

For the evaluation of the binding capability of SWCNTs **1a**–**c** toward individual nucleobase in ssDNA, each nanotube was separately hybridized with homo-oligomers ss-dA_25_, ss-dT_25_, and ss-dC_25_ under neutral conditions (pH = 7.4). Nine distinct hybrid complexes were thereby generated, and their binding efficiencies are shown in Figure 1(i)–(iii). Each figure set displayed three CD spectra corresponding to the hybrid complexes along with the spectrum of the parent 25-mer. Among the nine cases, six exhibited pronounced spectral changes relative to their unbound ssDNA controls. These include either distinct wavelength shifts (Δλ) or variations in molar ellipticity (θ), which indicate perturbations in the secondary structure of oligonucleotides. The relative binding strengths, derived from these spectral changes, are listed in columns 2–4 of Table 1. Binding efficiency was assessed on the basis of two criteria. First, the magnitude of Δλ was determined from shifts λ_max_ or λ_min_ of the CD, including bathochromic (longer wavelength) or hypsochromic (shorter wavelength) shifts. Second, the degree of ellipticity change was quantified; it reflects the extent of conformational alteration upon complexation.^12^

**Figure 1:**
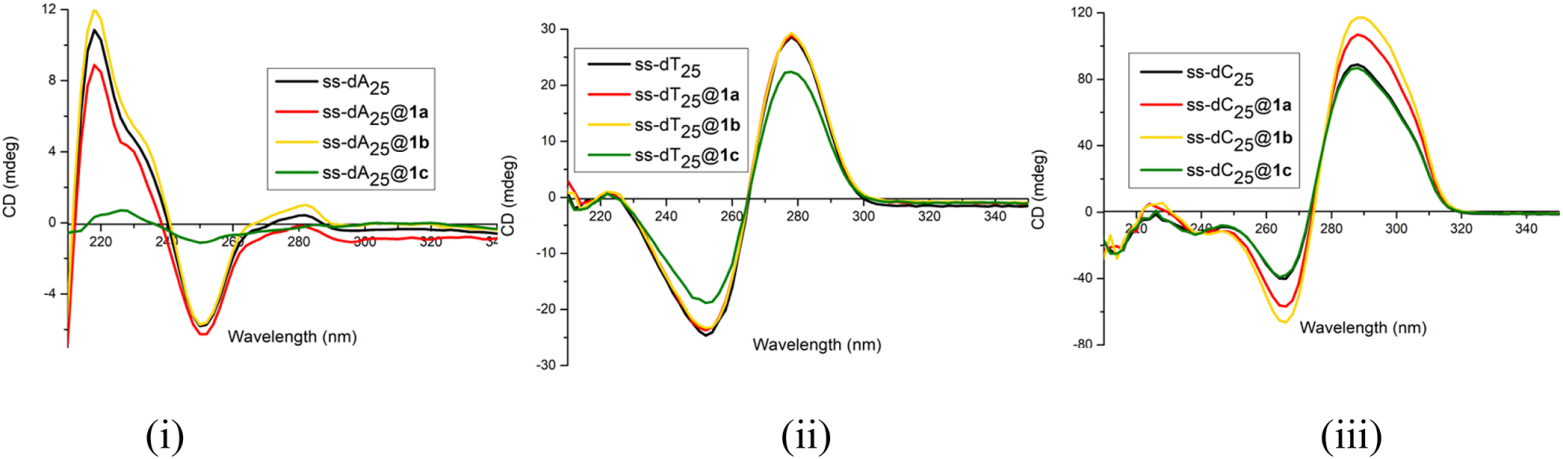
CD spectra of *f*-SWCNTs **1a**–**c** in buffer (pH 7.4, room temperature) entwined with (i) ss-dA_25_, (ii) ss-dT_25_, and (iii) ss-dC_25_. Each panel includes the corresponding parent oligomer as a reference.

**Table 1:**
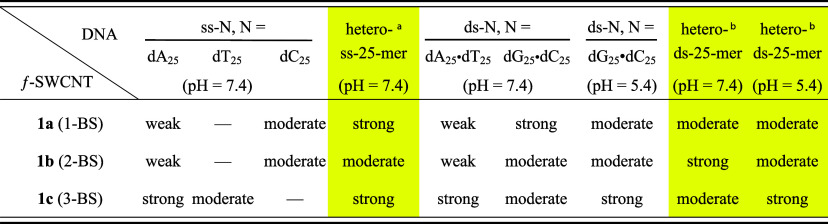
Evaluation of Hybridization Strength between SWCNTs **1a**–**c** Bearing Homogeneous Five-Membered Insertors with 25-mer ss- and ds-DNAs^a^ ^b^

a With a sequence of 5′-dTCGAGTACGTCGCCGTCCAGCTCGA-3′.

b ds-DNA formed by the above sequence and its complementary strand.

Our CD results in Figure 1(i)–(iii) demonstrate distinct binding preferences of *f*-SWCNTs **1a**–**1c** toward homo-oligomeric ssDNAs. Both *f*-SWCNTs **1a** (one binding site) and **1b** (two binding sites exhibited weak binding capability with ss-dA_25_ and moderate binding with ss-dC_25_ but showed no detectable interaction with ss-dT_25_ (Table 1, columns 2–4). In contrast, *f*-SWCNT **1c**, bearing three binding sites, displayed strong binding toward ss-dA_25_ and moderate binding toward ss-dT_25_, but no binding to ss-dC_25_. From the DNA perspective, ss-dA_25_ strongly bound to **1c** but only weakly bound to **1a** and **1b** (Figure 1(i) and column 2 of Table 1). ss-dT_25_ moderately bound to **1c** while no binding was observed with **1a** and **1b** (Figure 1(ii) and column 3). Conversely, ss-dC_25_ moderately bound to **1a** and **1b** but not to **1c** (Figure 1(iii) and column 4). Collectively, these results establish that *f*-SWCNT **1c** not only surpasses **1a** and **1b** in overall binding strength toward ssDNA but also displays clear base selectivity with strong affinity for adenine-rich sequences, moderate interaction with thymine, and poor affinity for cytosine.

Natural DNA consists of two polynucleotide strands held together by base pairing to form a double helix. According to Chargaff’s base-pairing rules,^13^ purine nucleobases complement pyrimidines via hydrogen bonding: A pairs with T and G pairs with C, as shown in Figure 2(i) and (ii). In this study, we modified the thymine (T) base at its carbonyldiamide moiety with different five-membered heterocycles: imidazolidin-2-one in **1a**, imidazolidine-2,4-dione in **1b**, and urazole in **1c**. These heterocycles act as artificial nucleobases and were found to form hydrogen bonds with adenine residues in DNA (e.g., **1c** in Figure 2(iii)).

The *N*-HDBs in *f*-SWCNT **1a** contain a single HN–C=O group available to form hydrogen bonding with adenine, whereas **1b** carries two HN–C=O groups fused together. In contrast, *f*-SWCNT **1c** possesses two spatially separated HN–C=O groups. This structural feature likely accounts for **1c** exhibiting the strongest binding affinity toward dA_25_ among **1a**–**c**. A similar interaction occurs between adenine (A) in ssDNA with thymine (T) in its complementary strand (see Figure 2(i)), as well as between adenine and urazole in *f*-SWCNT **1c** (see Figure 2(iii)).

**Figure 2:**
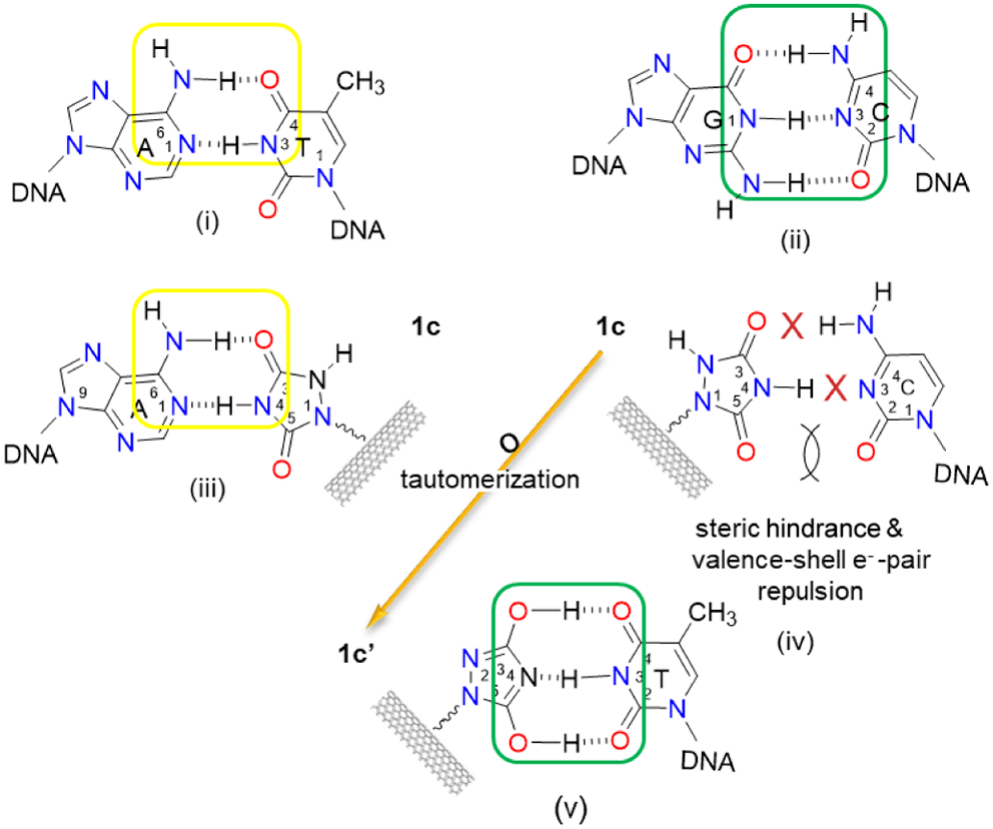
Established base-pairing: adenine–thymine (i) and guanine–cytosine (ii); possible base pairing: adenine–urazole (iii) and urazole–thymine (v). Electron-pair repulsion between urazole and cytosine is illustrated in (iv).

Cytosine (C) contains an NH_2_ group at its C_4_ position and can form hydrogen bonds with **1a** and **1b**, but not with **1c**. This may be attributed to valence-shell electron-pair repulsion between the C=O group at the C_2_ position of cytosine and the C=O group at the C_5_ position of the urazole in **1c** (see Figure 2(iv)). In addition, the urazole moieties in **1c** can undergo tautomerization.^14^ Consequently, *f*-SWCNT **1c’** is capable of forming three hydrogen bonds with thymine (T), as displayed in Figure 2(v). This phenomenon is analogous to the canonical Watson–Crick base pairing in normal dsDNA.^14,15^

### Hybridization of ds-Oligomers with *f*-SWCNTs

To investigate the bonding strength and binding mode in triplex-DNA formation, we hybridized SWCNTs **1a**–**c** individually with two ds-DNAs, comprising dA_25_•dT_25_ and dG_25_•dC_25_, under neutral conditions (pH = 7.4). Circular dichroism (CD) spectra shown in Figure 3 reveal that *f*-SWCNT **1a** exhibited weak binding to dA_25_•dT_25_ but strong binding to dG_25_•dC_25_; **1b** showed weak binding to dA_25_•dT_25_ and moderate binding to dG_25_•dC_25_; whereas **1c** displayed strong binding to dA_25_•dT_25_ and moderate binding to dG_25_•dC_25_. Reciprocally, dA_25_•dT_25_ bound weakly to *f*-SWCNTs **1a** and **1b**, but strongly to **1c** (see Figure 3(i) and Table 1, column 6). Although dG_25_•dC_25_ bound to all *f*-SWCNTs, its interaction with *f*-SWCNT **1a** was stronger than that with **1b** and **1c** (Figure 3(ii) and Table 1, column 7).

**Figure 3:**
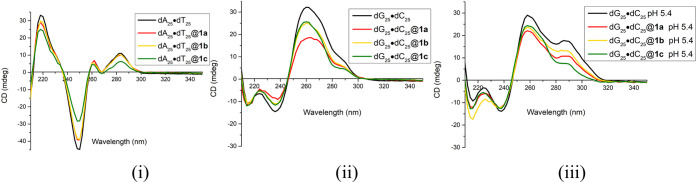
CD spectra of *f*-SWCNTs **1a**–**c** in buffer at 15 °C, entwined with (i) dA_25_•dT_25_ (pH 7.4), (ii) dG_25_•dC_25_ (pH 7.4), and (iii) dG_25_•dC_25_ (pH 5.4). Each set includes the corresponding parent duplex oligonucleotide as a reference.

To examine the effect of pH, we further hybridized *f*-SWCNTs **1a**–**c** with dG_25_•dC_25_ in buffer at pH 5.4 and 15 °C. As shown in the CD spectra of Figure 3 (iii), *f*-SWCNTs **1a** and **1b** exhibited moderate binding to dG_25_•dC_25_, whereas **1c** displayed strong binding (cf. λ_max_ = 288 nm). Under acidic conditions, the binding affinity followed the order **1c** > **1a** ≈ **1b** (column 8 in Table 1), which differs from that observed under neutral conditions, where the order was **1a** > **1b** ≈ **1c** (column 7).

Previously, *f*-SWCNTs **1a**–**c** were hybridized with dsDNA to form pseudo triplexes.^1^ The dsDNA was a hetero-25-mer with the sequence 5′-dTCGAGTACGTCGCCGTCCAGCTCGA-3′ annealed to its complementary strand. Under neutral conditions, the binding affinity followed the order **1b** > **1a** ≈ **1c** (Figure 4(i); Table 1, column 9).^1^ In contrast, under acidic conditions, **1c** exhibited the strongest binding compared with **1a** and **1b** (Figure 4(ii); Table 1, column 10). These observations are consistent with the formation of stable, genuine triplex DNAs that contain cytidines in the third oligopyrimidine strand, which preferentially form under slightly acidic conditions (pH 5.0–5.5).^16–19^

**Figure 4:**
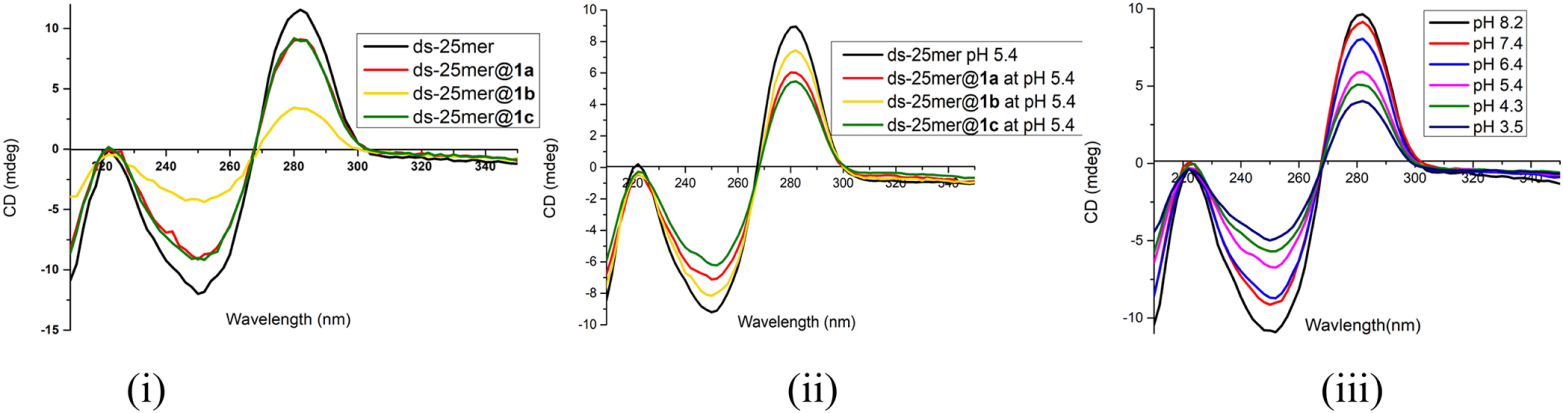
CD spectra of *f*-SWCNTs **1a**–**c** entwined with dsDNA (heterods-25-mer) under different conditions: (i) pH 7.4 at 25 °C,^1^ (ii) pH 5.4 at 15 °C, and (iii) *f*-SWCNT **1c** entwined with dsDNA at various pH (3.5–8.2).

Overall, *f*-SWCNT **1c** exhibited the strongest binding among **1a**–**c** toward most polynucleotides listed in Table 1. Accordingly, we performed CD experiments to examine the effect of pH on the formation of triplex dsDNA@*f*-SWCNT **1c**. As shown in Figure 4(iii), the intensity of the CD peaks decreased progressively as the pH of the PBS buffer was lowered from 8.2 (adjusted with 1.0 M NaOH_(aq)_) by using 1.0 M HCl_(aq)_. A pronounced drop in intensity occurred at pH 5.4 (pink line). At pH 3.5, the λ_max_ (dark iron curve) remained at 282 nm, identical to the parent λ_max_ observed at pH 8.2. Furthermore, no precipitation of *f*-SWCNT **1c** was detected under these pH conditions at 25 °C after 24 h.

### Binding Modes of ds-Oligomers with *f*-SWCNTs **1a–c**

The structural characteristics of DNA binders **2a**–**c** in *f*-SWCNTs **1** resembled those of thymine (T): all contained one heterocyclic ring and a common N–C­(=O)–NH or N–C­(=O)–NH–C­(=O) moiety (i.e., **1a** and **1b**, respectively). Accordingly, they could potentially form triplex structures of the type (binder)_*n*_–(A•T)_*n*_ through interactions in the major groove. Notably, the urazole moieties in **1c** share an extended structural motif with thymine, N–C­(=O)–NH–C­(=O)–NH, which underlines their strong affinity for A•T base pairs (Figure 5(i)). With three binding sites, the urazole units of **1c** are particularly well suited to penetrate the major groove of dsDNA. Consequently, urazole-grafted nanotubes **1c** displayed a stronger binding affinity than **1a** (one binding site) and **1b** (two binding sites), in agreement with their CD spectra shown in Figure 3(i).

**Figure 5:**
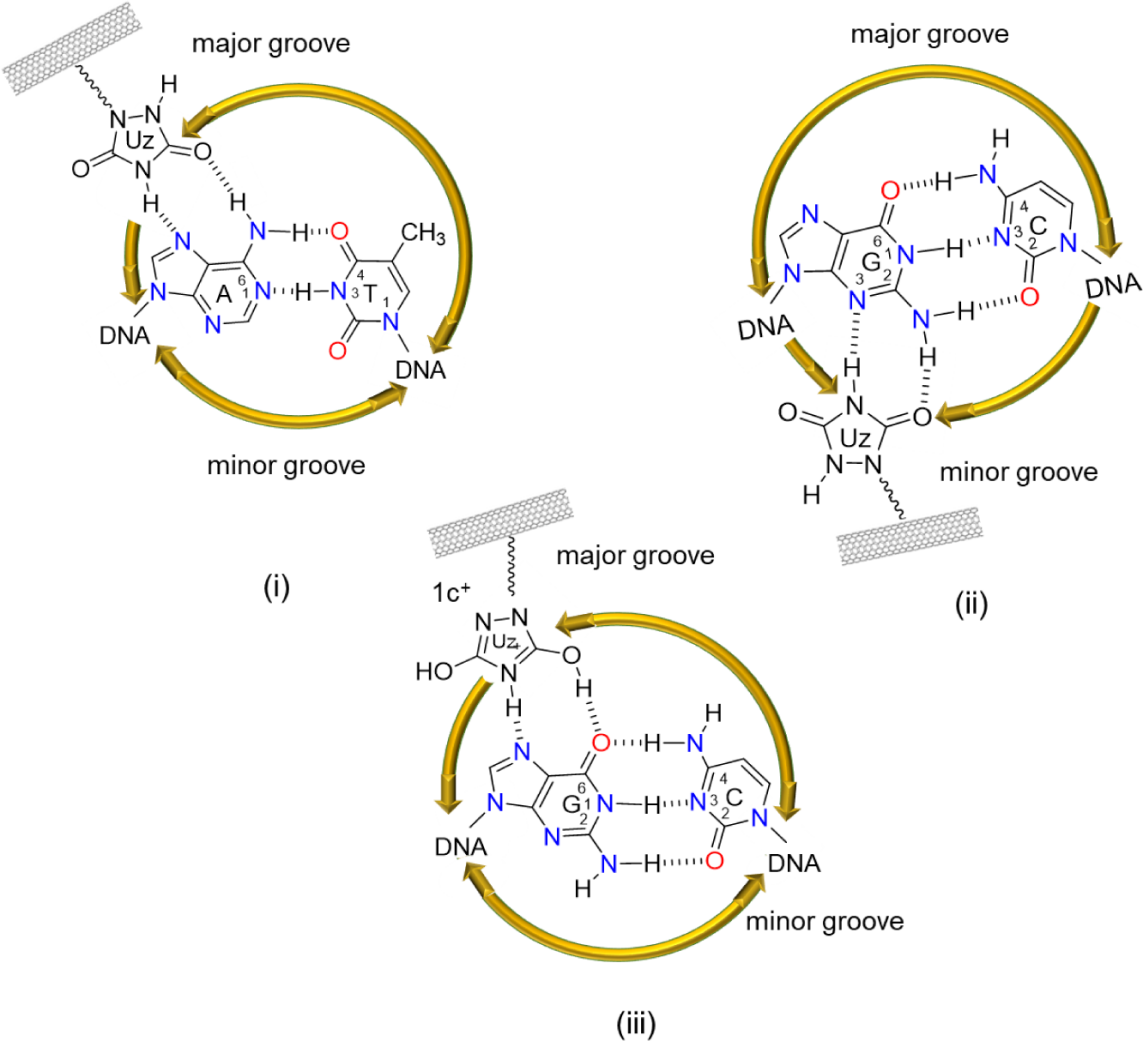
Possible binding modes for triplex formation between urazole in *f*-SWCNT **1c** and (homopurine•homopyrimidine) duplexes include: (i) urazole–(A•T), (ii) urazole–(G•C) under neutral conditions (pH 7.0), and (iii) protonated urazole^+^–(G•C) under acidic conditions (pH 5.4).

Two possible binding modes of (urazoles-**1c**)_*n*_–(G•C)_*n*_ pseudo triplexes are shown in Figure 5(ii) and (iii). At pH 7.4, hydrogen bonding between *f*-SWCNTs **1** and (G•C)_*n*_ is likely mediated through the minor groove. Although all three *f*-SWCNTs **1a**–**c** formed pseudo triplexes with (G•C)_*n*_ (see Figure 3(ii)), *f*-SWCNT **1a** displayed the strongest binding capability. This can be attributed to the imidazolidin-2-one unit in **1a** with a smaller size and fewer carbonyl groups. As a result, it is more compatible with the confined geometry of the minor groove than the larger binders in **1b** and **1c**.

On the other hand, *f*-SWCNT **1c** exhibited the strongest binding capability when the medium was acidified (pH = 5.4, Figure 3(iii)). Nucleobase protonation generally follows patterns dictated by their p*K*_a_ values.^14^ Urazole (**1c**) has a p*K*_a_ of 5.8, while 1-substituted urazoles have p*K*_a_ values in the range of 4.8–5.3 in water.^20a^ These values are comparable to that of protonated cytosine (C^+^, p*K*_a_ ≈ 4.2–4.5).^20b,20c^ Thus, under sufficiently acidic conditions, protonated urazole can mimic C^+^ and promote triplex formation with (G•C) pairs through the major groove, as illustrated in Figure 5(iii).^17,19^ By contrast, imidazolidin-2-one (**1a,** p*K*_a_ ≈ 14.58)^20d^ and hydantoin (**1b,** p*K*_a_ ≈ 9.16)^20d^ do not exhibit such protonation behavior and therefore lack this binding feature.

The linear decrease in the intensity of the positive band with decreasing pH can be attributed to the protonation of the amide groups of the base pairs. Such a process alters the geometrical features of the helical structures.^12^

The protonation of nucleobases and urazole derivatives may also deviate from their bulk-solution p*K*_a_ values due to factors such as electrostatic shielding by nearby phosphate groups, partial dehydration within the DNA grooves, and the proximity of neighboring heteroatoms. These local effects can stabilize protonated species at pH values slightly higher than those predicted from bulk-solution measurements.^21^ Accordingly, the enhanced binding affinity of urazole-functionalized **1c** observed at pH 5.4 is likely a consequence of both protonation-driven hydrogen bonding and microenvironment-facilitated interactions within the major groove.^22^

At neutral pH, **1a** is compact and uncharged, allowing it to insert into the minor groove (∼5–6 Å) without significant distortion of B-DNA geometry. The imidazolidinone headgroup of **1a** positions its carbonyl (O) and NH donor toward the G≡C edge, which is consistent with Hoogsteen-type hydrogen bonding. This explains the preferential binding of **1a** to G/C-rich duplexes.^23^ At mildly acidic pH (∼5.4), the urazole unit of **1c** becomes protonated at N^2^ and forms the cationic urazolium species (**1c** ^**+**^). Protonation increases steric bulk and electropositive character, which directs **1c** ^+^ toward the major groove (∼11–12 Å). The major groove can accommodate **1c** ^+^ and provides complementary electronegative sites (C^6^=O and N^7^ of G). This accounts for the observed inversion of groove-binding selectivity.^23^

Protonation also enhances binding affinity through electrostatic interactions with dsDNA and substantially reduces the overall negative charge of the phosphate backbones.^24–26^ For further investigation of these binding characteristics, advanced 3D NMR experiments will be conducted in the future. It will involve the use of doubly labeled (^13^C and ^15^N) binders.

### Synthesis of Shortened *f*-SWCNT **1c**

By modifying the established procedures reported by Smalley,^27^ Peng,^28^ Fagan,^29^ and their coworkers, we first shortened commercially available pristine SWCNTs of lengths (5000–30 000 nm) through the sequential steps (i) → (ii)→ (iii) shown in Scheme 1. Separation of the resultant mixture (step (iii) → (iv)) afforded short SWCNTs with lengths of 40–350 nm. These nanotubes were then functionalized via steps (iv)–(vi) to afford the target **1c**.

**Scheme 1:**
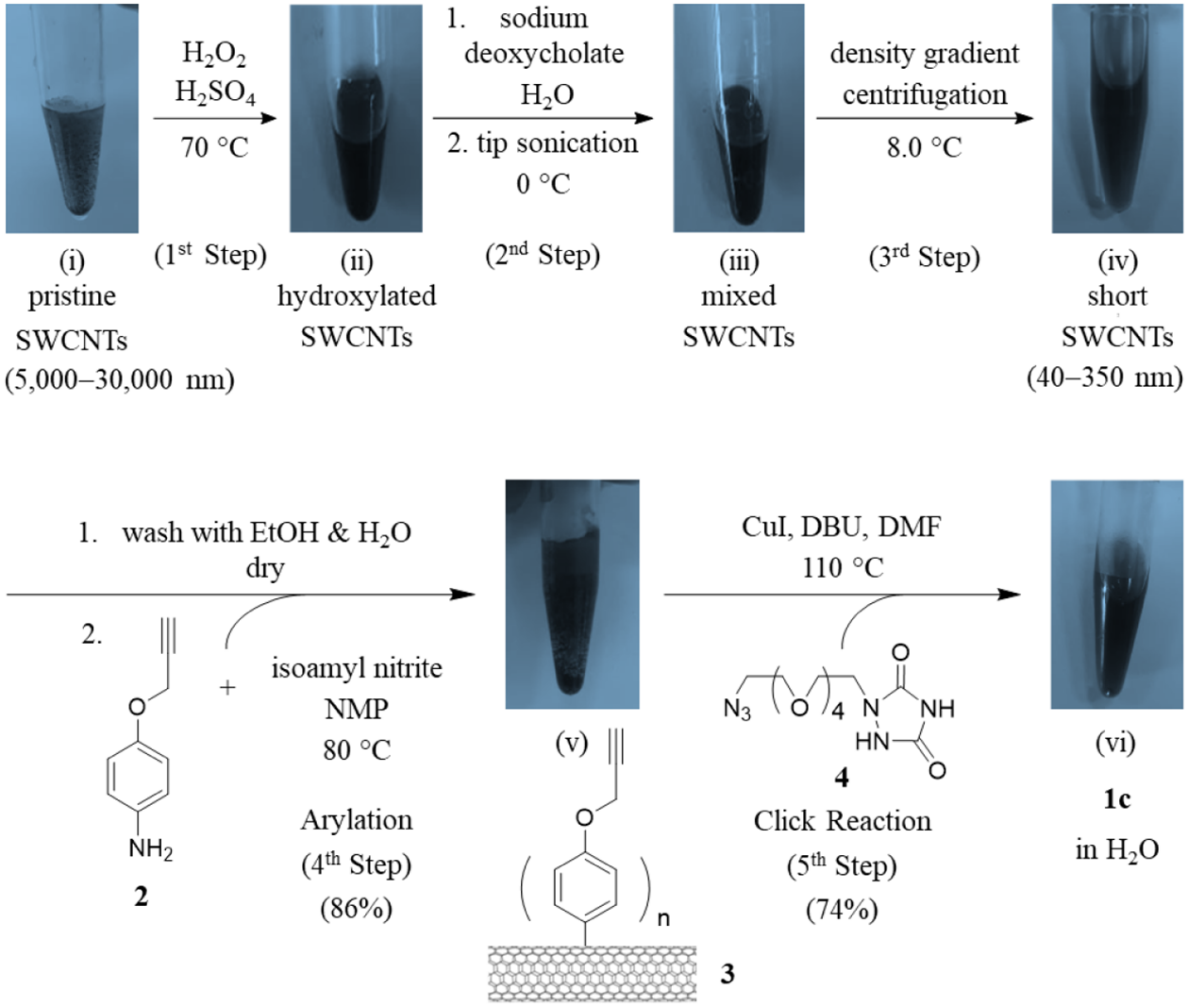
Cleavage of Long Pristine SWCNTs and Synthesis of Short *f*-SWCNT **1c** ^a^

a The overall process consisted of five steps: Steps 1, 4, and 5 were chemical transformations, whereas Steps 2 and 3 involved separation procedures.

The pristine SWCNTs in vial (i) were first treated with Piranha solution^25^ (a mixture of sulfuric acid, water, and hydrogen peroxide) at 70 °C for 7.0 h to introduce hydroxyl functionalities on the nanotube surfaces.^30^ The resultant hydroxylated SWCNTs in vial (ii) were then dispersed in aqueous sodium deoxycholate^31^ and subjected to tip sonication (on/off cycles at 50% amplitude, 20-s-cycle without autoclaving) to cut the nanotubes into smaller fragments.^28,32^ Sonication for 4.0 h was sufficient to generate a large number of fragments in vial (iii). The mixture of shortened nanotubes was subsequently separated by density gradient centrifugation.^29^ Optimal separation was achieved at 12 000 rpm at 8 °C for 96 h; lower temperatures should be avoided, as they may cause the solution to freeze. The bottom fractions were collected, transferred into 90% ethanol in water, and left standing for 24 h to precipitate the desired short SWCNTs in vial (iv), with lengths of 40–350 nm. The black charcoal-like SWCNTs were collected and washed with 70% ethanol (×2) and deionized water (×2), and the resultant brownish-black residues were dried over P_2_O_5_ powder under vacuum. Atomic force microscope (AFM) was used to confirm the formation of the short SWCNTs (Figure 6(i)), and their length distribution (<350 nm) is shown in Figure 6(ii).

**Figure 6:**
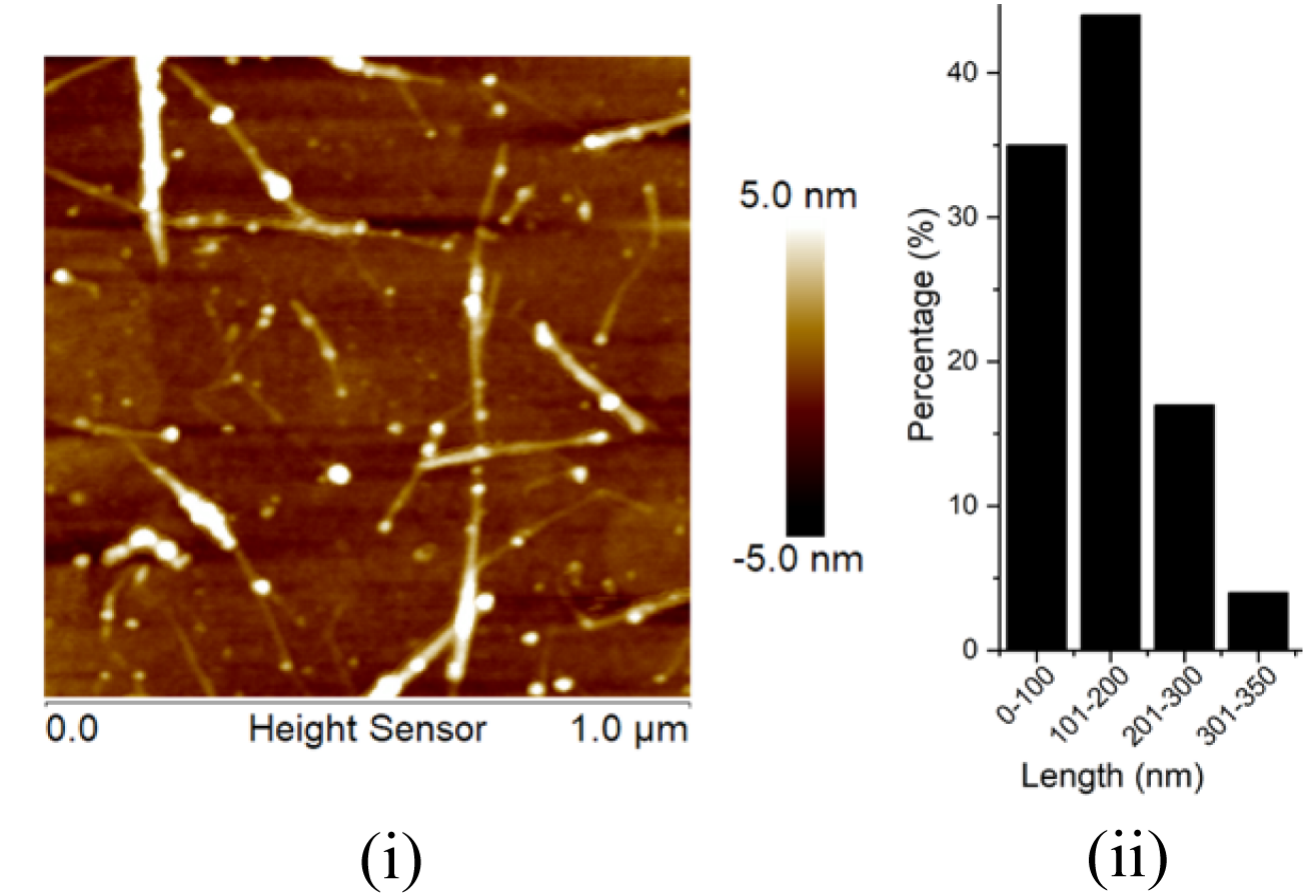
(i) AFM image of the shortened SWCNTs and (ii) corresponding length distribution.

The separated and shortened pristine SWCNTs in vial (iv) of Scheme 1 were grafted with ligand **4**, which contains urazole as the DNA-binding moiety, by following our established procedure.^33^ This involved an arylation reaction of the shortened pristine SWCNTs with alkynyl aniline **2** in the presence of isoamyl nitrite in *N*-methyl-2-pyrrolidone (NMP), as shown in the fourth equation step. The resultant short *f*-SWCNT **3**, bearing multiple (phenoxy)­alkynyl ligands^34^ (vial (v)), was obtained in 86% yield. Subsequently, a click reaction was performed between SWCNTs **3** and urazole-containing azide **4** ^33^ in the presence of CuI and diazabicycloundec-7-ene (DBU) in DMF. This afforded the desired short *f*-SWCNT **1c** in vial (vi) with a 74% yield in the fifth step. Each (phenoxy)­alkynyl ligand in *f*-SWCNT **1c** corresponds to an average of ∼41 carbon atoms per ligand along the central nanotube framework.

### *In Vivo* Toxicity Assessment of Short *f-*SWCNT **1c** against *Caenorhabditis elegans* (*C. elegans*)

The target short *f-*SWCNT **1c** were subjected to an *in vivo* toxicity evaluation against *C. elegans*, a widely adopted model for the assessment of drugs and chemicals in biomedical research. The *C. elegans* assay is demonstrated as a reliable, rapid, and cost-effective platform compared with conventional cell-culture systems for the evaluation of potential toxicity at both the organismal and molecular levels relevant to human health.^35^ It is also commonly employed to study the toxicity of environmental contaminants, including metals, fine particulate matter, and so forth.^36,37^ Recently, *C. elegans* has increasingly been used as a model for the investigation of the toxicity of nanomaterials.^38^

To assess the ecotoxicity of *f*-SWCNT **1c** on nematodes, we treated *C. elegans* with short *f-*SWCNT **1c** at concentrations of 50, 100, and 250 μg/mL. The thrashing assay, a standard method for the evaluation of locomotor deficits associated with motor neuron dysfunction, was performed. Motor neuron defects often serve as hallmarks of neurological disorders, such as amyotrophic lateral sclerosis (ALS) and Parkinson’s disease.^39–41^ As shown in Figure 7(A), treatment with short **1c** at concentrations of 100 μg/mL or higher resulted in impaired motor neurons. The thrashing phenotype is consistent with motor circuit perturbation. However, alternative contributors likely include muscle integrity and neuromuscular junction (NMJ) function (e.g., altered cholinergic signaling and postsynaptic sensitivity), energy/metabolic status (ATP dependence and mitochondrial dysfunction), and generalized stress responses that suppress locomotion without direct neuronal damage.^42–46^

**Figure 7:**
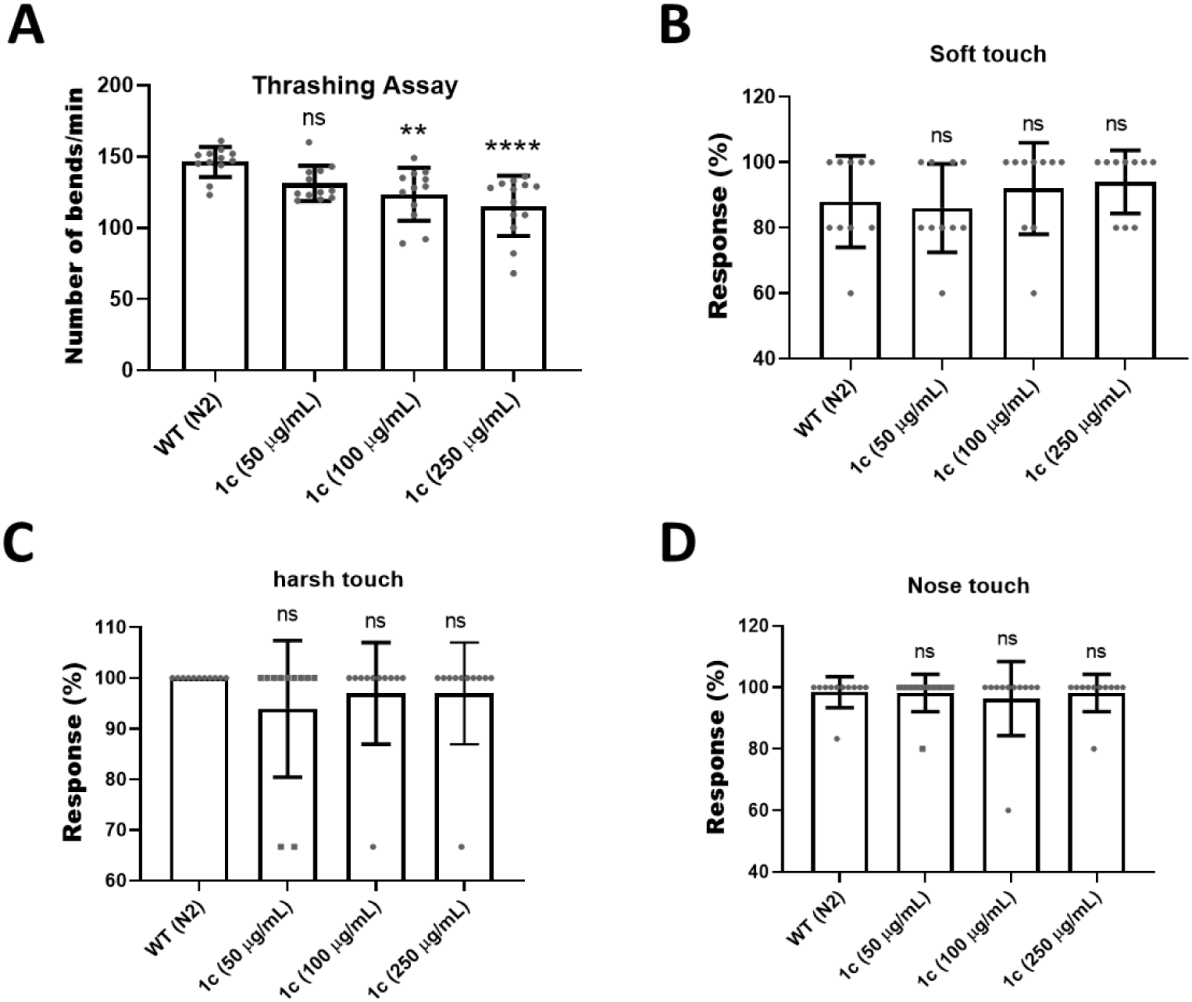
Effect of *f*-SWCNT **1c** on *C. elegans* behavior. (A) Thrashing assay (body bends per minute in M9 buffer), *n* = 12 worms. (B) Soft touch response analysis (*n* = 12 worms). (C) Harsh touch response analysis (*n* = 12 worms). (D) Nose touch response analysis (*n* = 12 worms). WT (N2) denotes the wild-type N2 strain. Error bars represent ±SD; ns = nonsignificant. Statistical analysis was performed using one-way ANOVA followed by Dunnett’s test. *p < 0.05, **p < 0.005, ***p < 0.001, ****p < 0.0001.

Sensory neuron function was then assessed by using touch assays to evaluate mechano- and chemosensation. In *C. elegans*, distinct sets of neurons mediate responses to different types of touch, including soft, harsh, and nose-specific stimuli. Touching the nose or anterior body region induces backward movement, known as the MEC response. Soft touch was applied by touching the worm with an eyelash, whereas harsh touch was delivered with a platinum wire.^47^ Our results showed no significant differences across the different concentrations of **1c**. These findings indicate that *f*-SWCNT **1c** was nontoxic to sensory neurons (Figure 7(B)–(D)).

*C. elegans* is a small (∼1 mm in length) transparent nematode with a short life cycle (∼3.5 days) and a fully sequenced genome.^48,49^ Previous studies have demonstrated that *C. elegans* is an effective model to evaluate nanoparticle toxicity.^40,50–52^ Our results indicate that *f*-SWCNT **1c** is nontoxic to sensory neurons. These results were consistent with prior reports showing that SWCNTs do not induce acute toxicity in *C. elegans* suspending in Cys–SWNT solutions at concentrations of 50, 100, and 250 μg/mL.^53^ However, we observed impaired motor neuron function when worms were treated with *f*-SWCNT **1c** at concentrations of 100 μg/mL and above (Figure 7(A)). Previous articles reported that multiwalled carbon nanotubes (MWCNTs) exert toxic effects on *C. elegans* motor neurons at concentrations >0.01 ng/mL, whereas MWCNTs–COOH cause toxicity at concentrations >0.1 μg/mL.^54^ Therefore, *f*-SWCNT **1c** is approximately 10^7^ times less toxic than MWCNTs and 10^3^ times less toxic than MWCNTs–COOH. These results reflect relative safety under our specific test conditions. However, toxicity values derive from disparate assay formats, material properties (length, diameter, surface functionalization), exposure regimes, end points, and organismal contexts.

Motor impairment observed at higher *f*-SWCNT **1c** doses can be reconciled with reports of generally nontoxic SWCNTs by considering several factors: (i) concentration thresholds, as effects emerge at ≥100 μg/mL; (ii) functionalization, where urazole/PEG modification enhances dispersibility and reduces aggregation-related systemic toxicity but may increase localized interactions at high doses; (iii) length-dependent uptake, since shortened 40–350 nm SWCNTs are more readily internalized, leading to greater local bioactivity; and (iv) assay sensitivity, as *C. elegans* locomotion—driven by >100 motor neurons—is highly responsive to subtle neuromuscular perturbations.

While *f*-SWCNT **1c** affected nematode thrashing, it did not impair mechano- or chemosensation. The thrashing assay is a standard method to assess motor neuron defects, whereas touch assays evaluate sensory neuron function. In *C. elegans*, only two neurons (PVC and PVD) mediate harsh touch^55,56^ and four neurons (ALM, AVM, PLM, and PVM) mediate soft touch.^57^ In contrast, *C. elegans* possesses over 100 motor neurons. Thus, from a statistical perspective, it is likely that *f*-SWCNT **1c** affected some motor neurons rather than the limited number of sensory neurons.

## Conclusions

Carbon nanotubes (CNTs) are inherently rigid, strong, and stiff materials. After the pristine SWCNT was grafted with small five-membered heterocycles through flexible and amphipathic spacers, all three *f*-SWCNTs **1a**–**c** were able to hybridize with the majority of ss- and ds-DNAs. The heterocyclic moieties—imidazolidin-2-one, hydantoin, and urazole—functioned as “artificial nucleobases” with one, two, and three binding sites, respectively. Among them, *f*-SWCNT **1c** containing homogeneous urazole moieties with three binding sites often exhibited strong binding capability. Notably, dA and dT in ssDNA, as well as d­(A•T) and d­(G•C) under acidic conditions in dsDNA, preferentially bound to *f*-SWCNT **1c** over *f*-SWCNTs **1a** and **1b**, whereas dC showed less binding affinity.

In the pseudotriplexes, the central poly­(fused cyclohexanes) networks of *f*-SWCNTs **1a**–**c** retained their rigidity. Unlike conventional third-stranded oligonucleotides, which are highly flexible, the rigid *f*-SWCNTs **1a**–**c** could function as “pseudo third strands” due to the flexible long organic ligands attached to the central networks. This design allowed the five-membered heterocyclic moieties to reach the nucleobases of dsDNA effectively and form hydrogen bonds. Consequently, the duplex targeting during triplex formation is no longer restricted to a homopurine–homopyrimidine sequence on one strand of a normal duplex.^16^ *Triple-helical structures can thus be formed by use of any of the f-SWCNTs* **1a** *–* **c** *as artificial pseudo third-stranded DNA.* Table 1 summarizes guidelines for the application of *f*-SWCNTs **1a**–**c** to hybridize with ss- and dsDNAs to form pseudo duplex-like and pseudo triplex-like DNAs, respectively.

For the minimization of potential risks associated with long carbon nanotubes, short *f*-SWCNT **1c** with lengths of 40–350 nm was prepared. Its *in vivo* toxicity was evaluated by using *C. elegans* through soft, harsh, and nose-touch assays. Our results indicate that *f*-SWCNT **1c** is safe for *C. elegans* at relevant concentrations, although it exhibits toxic effects on motor neurons at concentrations ≥100 μg/mL. This study further underscores the utility of *C. elegans* as a powerful model for the evaluation of the toxicity of nanomaterials.

## Methods

### Circular Dichroism (CD) Measurement

The CD spectra of DNA hybrids in a sodium phosphate buffer were recorded on a CD spectrometer (Model 410, AVIV Biomedical Inc.). Before measurement, the samples were placed in rectangular quartz glass cuvettes (Hellma GmbH & Co. KG) with a 1.00 mm optical path length at 25 °C. To prevent ozone formation and minimize damage to the optical system, the instrument was purged with N_2_ gas to remove O_2_ from the lamp housing and sample compartment. DNA hybrids (0.40 mL each) were pipetted into the cuvette, and measurements were taken over a spectral range of 400 to 200 nm, with a scan rate of 10.0 nm/min and a spectral width of 2.00 nm. Additionally, ssDNA and dsDNA were measured for comparison. All data were analyzed by using Aviv Biomedical Inc. and Origin software.

### Atomic Force Microscopy (AFM) Measurement and Sample Preparations

Tapping-mode atomic force microscopy (AFM) measurements were performed in air using a Bruker Dimension Icon system (Bruker Instruments). The system was operated under ambient conditions with phosphorus-doped silicon tips (Olympus OMCL, with a resistivity of 0.01–0.02 Ohm/cm, a tip length of 160 μm, a width of 30.0 μm, a normal spring constant of 26 N/m, and a resonance frequency in the range of 240 to 300 kHz). The AFM provided a spatial resolution of *xy* <0.150 nm and *z* ∼0.035 nm. For sample preparation, a droplet of poly-l-lysine solution (1.0%), freshly diluted with deionized water, was deposited onto freshly cleaved mica (muscovite) and incubated for 15 min. The surface was then extensively rinsed with water and dried under nitrogen. Short, functionalized SWCNT **1c** samples were dispersed in water at a concentration of 5.0 μg/mL, deposited onto silicon wafers (2.00 × 2.00 mm), and dried at room temperature. Images were analyzed using NanoScope Analysis and ImageJ software.

### Nematode Behavioral Assays

Worm strains were maintained at 20 °C on nematode growth medium agar plates seeded with the uracil auxotroph *E. coli* OP50 strains, serving as the food source in accordance with standard protocols.^48^ All analyses in this study were performed on young adult worms (Figure 8). Worm strain N2 (wild type) was obtained from the *Caenorhabditis* Genetics Center (CGC, MN, USA). Synchronized worms were exposed to the nanomaterial DL-Cut CNT at concentrations of 50, 100, and 250 μg/mL, prepared in M9 buffer (3.0 g KH_2_PO_4_, 6.0 g Na_2_HPO_4_, 5.0 g NaCl, 1.0 mL of 1.0 M MgSO_4_, and distilled water to a final volume of 1.0 L, sterilized by autoclaving). Synchronized worms were exposed to the nanomaterial DL-Cut CNT at concentrations of 50, 100, and 250 μg/mL, prepared in M9 Buffer (3.0 g KH_2_PO_4_, 6.0 g Na_2_HPO_4_, 5.0 g NaCl, 1.0 mL of 1.0 M MgSO_4_, and H_2_O to a final volume of 1.0 L, sterilized by autoclaving). The *f*-SWCNT **1c** suspensions were visually inspected before and during the exposure period. They remained well dispersed and optically homogeneous in the worm medium over the assay period, with no visible aggregation or precipitation observed. Thrashing frequency was determined as the worms’ body bends per minute.^58^ For the nose and soft touch response, each worm was subjected to five touches. For the harsh touch assay, each worm was subjected to three touches. The worm’s responses were converted into percentages for data plotting.

**Figure 8:**
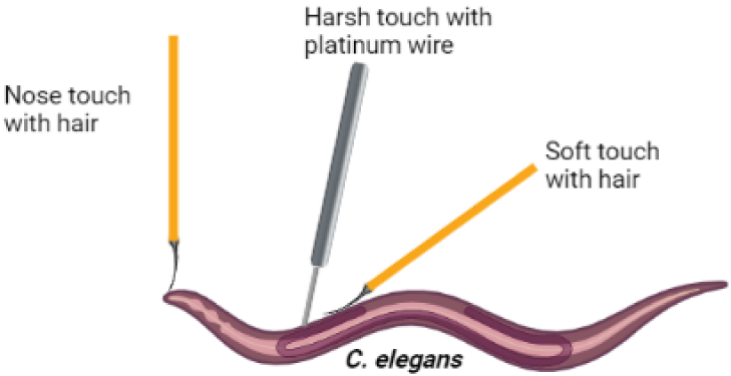
Representative depiction of nose-, harsh-, and soft-touch assays using young adult worms.

## Supplementary Material



## Notes

Preparation of Short Functionalized SWCNTs (*f*-SWCNTs); SEM Sample Preparation for Short SWCNT; Dynamic Light Scattering and Evidence; Nanotube-only and Pristine-SWCNT Control Experiments; Figure S1: SEM images of long and short SWCNTs; Figure S2: Size distribution of DNA@urazole *f*-SWCNT; Figure S3: Circular dichroism spectra of control and *f*-SWCNT systems; Figure S4: Circular dichroism spectra of ds­(G·C) duplexes in the absence and presence of urazole ligand; Table S1: Average hydrodynamic diameter and polydispersity index (PDI) of *f*-SWCNTs and their DNA hybrid complexes (PDF)
