# Supplementary material for: Urazole-Functionalized Carbon Nanotubes as Artificial DNA Strands and Their In Vivo Toxicity
Source: ACS Nano. 2026 Jan 28;20(5):4193–203. doi: 10.1021/acsnano.5c16279 (PMC12895559; doi:10.1021/acsnano.5c16279)
Supplement: Supplementary file 1 [file nn5c16279_si_001.pdf]

Supporting Information

Urazole-Functionalized Carbon Nanotubes as Artificial DNA Strands and Their *In Vivo* Toxicity

Shwu-Chen Tsay,[1,2] Deepa R. Landge,[1,2] Wen-Chieh Huang,[1,2] Uttam Patil, [1,2] Jia-Cherng Horng,[1,2] Chun-Cheng Lin,[1,2] Yu-Chen Hu,[2,3] Syed N. Barmaver,[4] Oliver I. Wagner,[4] and Jih Ru Hwu[1,2,*]

[1] Department of Chemistry, National Tsing Hua University, Hsinchu 300, Taiwan

[2] Frontier Research Center on Fundamental and Applied Sciences of Matters, National Tsing Hua University, Hsinchu 300044, Taiwan

[3] Department of Chemical Engineering, National Tsing Hua University, Hsinchu 300044, Taiwan

[4] Institute of Molecular and Cellular Biology, Department of Life Science, National Tsing Hua University, Hsinchu 300044, Taiwan

Corresponding Author's email: jrhwu@mx.nthu.edu.tw

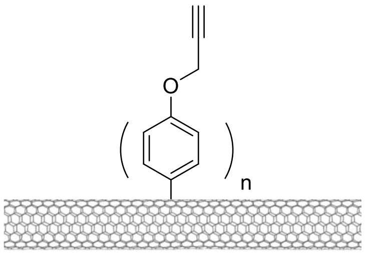

3

Preparation of Short Alkynated SWCNT 3.[1] Pristine single-walled carbon nanotubes (SWNTs) purchased from Chengdu Organic Chemicals Co. Ltd. were cut by use of the method reported by Smalley,[2] Peng,[3] and Fagan.[4] The SWCNTs (150.2 mg) were treated with the Piranha solution

(i.e., a mixture of sulfuric acid, water, and hydrogen peroxide) at 70 °C for 7.0 h to introduce hydroxyl groups onto the nanotube surfaces. The hydroxylated SWCNTs were then dispersed in an aqueous solution of sodium deoxycholate and subjected to tip sonication (50% amplitude, 20-second on/off cycles without autoclaving) to cut the nanotubes into shorter fragments. A sonication duration of 4.0 h was sufficient to afford a significant amount of SWCNTs, which were subsequently separated by density gradient centrifugation with a slower rate of 12,000 rpm at 8 °C for 96 h to improve resolution. Lower temperatures were avoided, as they caused the solution to freeze. The bottom fractions were collected and transferred into a 90% ethanol/water solution and left to stand for 24 h to precipitate the desired short SWCNTs with lengths ranging from 40 to 350 nm. The precipitated nanotubes, appearing as black charcoal-like material, were collected and washed with 70% ethanol (2 × 12 mL) and then deionized water (2 × 12 mL). The final product, a brownish-black residue, was dried under vacuum over powdered P_2O_5. The short SWCNTs (140.5 mg) were treated with 4-(prop-2-yn-1-yloxy)aniline (588 mg, 4.02 mmol, 4.2 equiv) in the presence of isoamyl nitrite (469 mg, 4.05 mmol, 4.0 equiv), and *N*-methylpyrrolidone (NMP, 15.0 mL) at 60 °C for 48 h. For elimination of the residual NMP, the retained material was cooled down and washed with CH_2Cl_2 (4 × 12 mL), and then dried under reduced pressure at 40 °C. The short alkylated SWCNT **3** was obtained as black powder (120.4 mg) in 86% yield.[1]

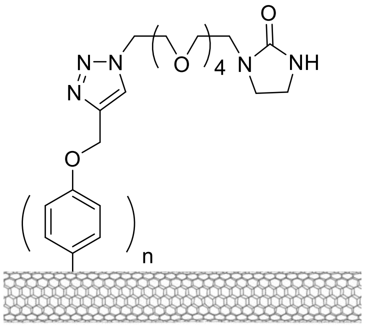

1a

Short Poly(imidazolidine-2-one)-SWCNT (1a). Short alkynated single-walled carbon nanotubes **3** (46.4 mg, corresponding to 1.01 mmol of carbon) were dispersed in dimethylformamide (DMF, 6.0 mL) by sonication at room temperature for 20 min. To the homogeneous black suspension was added azide 1-(14-azido-3,6,9,12-tetraoxatetradecyl)imidazolidin-2-one[5] (0.351 g, 1.06 mmol, 1.05 equiv), copper(I) iodide (CuI, 1.05 g, 5.56 mmol, 5.5 equiv), and 1,8-diazabicyclo[5.4.0]undec-7-ene (DBU, 2.49 g, 14.84 mmol, 14 equiv). The mixture was then heated to 110 °C and stirred continuously for 96 h. After cooling to ambient temperature, the resultant was diluted with THF (3 × 10 mL) and passed through a poly(tetrafluoroethylene) membrane filter with 450-nm pore size. The collected solid residue was washed extensively with DMF (5 × 10 mL) and subsequently with THF (3 × 10 mL) to remove soluble by-products. The resultant solids were re-dispersed in NMP (2.0 mL) by sonication for 10 min. After standing undisturbed for 2–3 min, the less-dispersible unreacted SWCNTs settled to the bottom. The supernatant containing the functionalized short material was carefully decanted and filtered again by use of a poly(tetrafluoroethene) (PTFE) membrane. For elimination of the residual NMP, the retained material was washed with CH_2Cl_2 (3 × 10 mL) and then dried under reduced pressure at 40 °C. The short SWCNT **1a** was obtained as black powder (34.2 mg) in 74% yield. Infrared spectroscopy (KBr pellet) confirmed the expected functional groups therein: a broad N–H stretch at 3297 cm^{-1}, medium alkyl C–H stretch at 2993 cm^{-1}, strong and medium carbonyl absorptions at 1797 and 1746 cm^{-1}, respectively, along with characteristic bands at 1626, 1337 (C–N), 1288, 1219, and 1093 cm^{-1}. All of these peaks are consistent with closely related SWCNT in the published paper.[5]

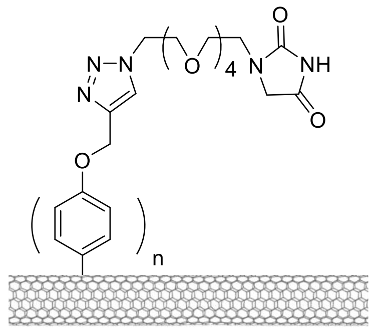

1b

Short (Poly(imidazolidine-2,4-dione))-SWCNT (1b). Short alkynylated SWCNT **3** (41.2 mg, corresponding to 1.02 mmol of carbon) was dispersed in DMF (10.2 mL) by sonication at room temperature for 10 minutes. To the resultant black suspension, azide 1-(14-azido-3,6,9,12-tetraoxatetradecyl)imidazolidine-2,4-dione[5] (0.346 g, 1.01 mmol, 1.05 equiv), CuI (1.05 g, 5.50 mmol, 5.5 equiv), and DBU (2.98 g, 14.1 mmol, 14 equiv) were added. The reaction mixture was stirred at 110 °C for 96 hours. The mixture was cooled to room temperature, diluted with THF (5 × 10 mL), and filtered through a PTFE membrane (pore size: 450 nm). The collected solids were washed sequentially with DMF (5 × 10 mL) and THF (5 × 10 mL). The resultant solid was then dispersed in NMP (2.0 mL) by sonication for 10 minutes. After standing for 2–3 minutes, the less dispersible alkynylated SWCNTs settled. The supernatant was carefully collected and filtered through a PTFE membrane. The retained solids were washed with CH_2Cl_2 (4 × 10 mL) to remove residual NMP and subsequently dried at 40 °C to afford the SWCNT **1b** (31.7 mg) as dark brown to black solids in 77% yield. IR: 3408 (br, N–H), 2924 (s), 2854 (m), 1728 (m, C=O), 1632 (m, C=O), 1463 (m), 1374 (w, C–N), 1260 (w), and 1166 (w). All of these peaks are consistent with closely related SWCNT in the published paper.[5]

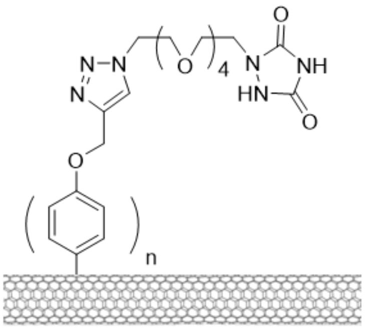

1c

Short (Polytriazolidine-3,5-dione)-Functionalized SWCNT (1c). Short alkynated single-walled carbon nanotubes **3** (47.4) mg, corresponding to 1.01 mmol of carbon) were dispersed in DMF (6.0 mL) by sonication at room temperature for 20 min. To the homogeneous black suspension was added azide 1-(14-azido-3,6,9,12-tetraoxatetradecyl)imidazolidine-2,4,5-trione[4,5] **4** (0.420 g, 1.21 mmol, 1.05 equiv), CuI (1.26 g, 6.65 mmol, 5.5 equiv), and DBU (2.57 g, 16.94 mmol, 14 equiv). The mixture was then heated to 110 °C and stirred continuously for 96 h. After cooling to ambient temperature, the resultant was diluted with THF (3 × 10 mL) and passed through a PTFE membrane filter with 450-nm pore size. The collected solid residue was extensively washed with DMF (5 × 10 mL) and subsequently with THF (3 × 10 mL) to remove soluble by-products. The resultant solids were re-dispersed in NMP (2.0 mL) by sonication for 10 min. After standing undisturbed for 2–3 min, the less-dispersible unreacted SWCNTs settled to the bottom. The supernatant containing the functionalized short material was carefully decanted and filtered again by use of a PTFE membrane. For elimination of the residual NMP, the retained material was washed with CH_2Cl_2 (3 × 10 mL) and then dried under reduced pressure at 40 °C. The short SWCNT **1c** was obtained as black powder (35.2 mg) in 74% yield. Infrared spectroscopy (KBr pellet) confirmed the expected functional groups therein: a broad NH stretch at 3552 cm^{-1}, medium alkyl C–H stretch at 2923 cm^{-1}, carbonyl absorptions at 1715 cm^{-1}, along with

characteristic bands at 1622, 1337 (C–N), 1455 weak, and 1115 cm^{-1}medium band. All of these peaks are consistent with closely related SWCNTs in the published paper.[5]

SEM Sample Preparation for Short SWCNT. The short SWCNT (0.102 mg) was sonicated for 10 min in aqueous solution (1.0 mL), which contained sodium deoxycholate (1.0 mg) in of deionized water (9.0 mL). After sonication, the sample (1.0 μL) was deposited on a silicon wafer Si/SiO_2 (2.00 mm × 2.00 mm) and dried at 30 °C. After drying, the same sample was washed in ethanol (70%, 5.0 mL) for 5 min. This procedure was repeated twice to remove the sodium deoxycholate, then the sample was wash with deionized (DI) water (5.0 mL) and dried at room temperature. The wafers were first coated with a thin layer (0.50 nm) of platinum by use of the sputtering technique (JEOL, JFC-1600 Auto fine coater) at 10.0 mA for 13 sec. SEM micrograph as presented in Figure S1 showed that the nanotubes were uniformly dispersed over a larger surface area and did not form bundles or aggregates. This independent morphological evidence supports the reliability of our AFM-derived length distribution and validates the effectiveness of the dispersion and functionalization procedures.[6]

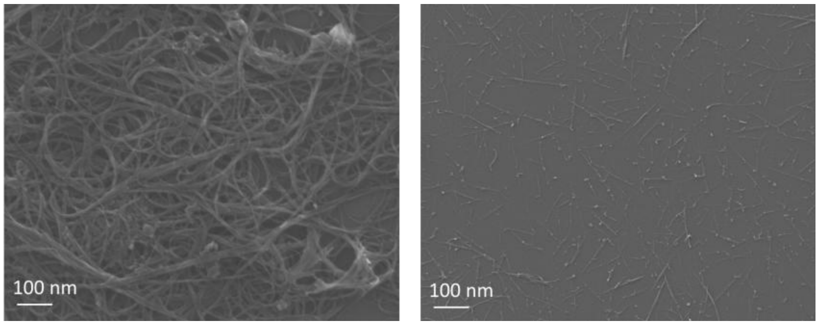

(i) (ii)

Figure S1. SEM images show (i) long tubes in tangled formed in the range of 1–5 μm before shortening; and (ii) well-separated short carbon nanotubes in the range of 30–380 nm after shortening.

Dynamic Light Scattering (DLS). DLS measurements conducted in the same aqueous medium used for spectroscopy indicate that functionalization and subsequent DNA hybridization afforded stable and uniform dispersions (see Figure S2):

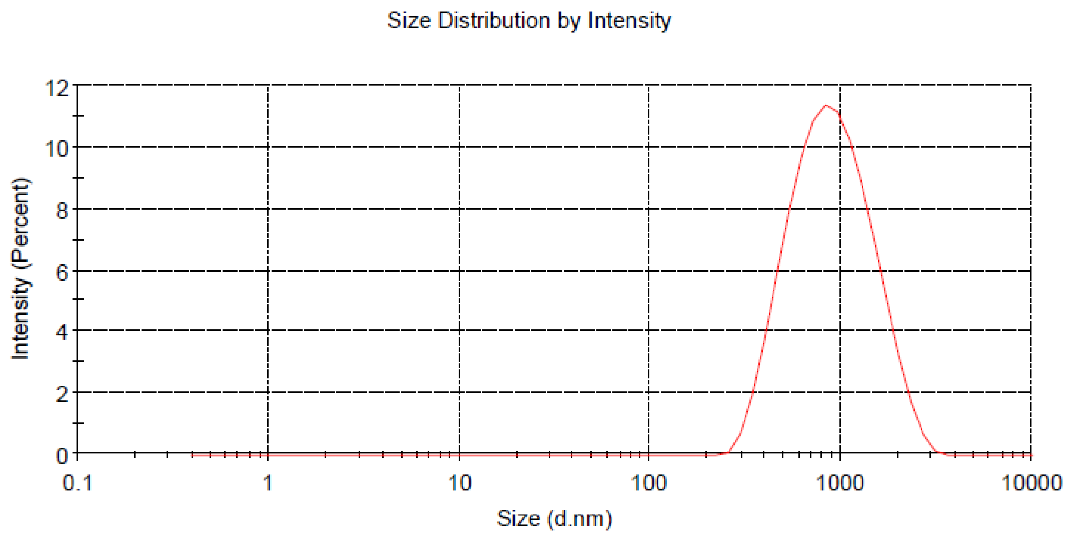

Figure S2. Size distribution shows that the hydrodynamic diameter of DNA@urazole functionalized SWCNT **1c** was approximately 980 nm in DI water at room temperature.

Table S1. Average hydrodynamic diameter and polydispersity index (PDI) of functionalized SWCNTs and their hybrid complexes in DI water.

Sample	Z-Average (d, nm):	PDI
Arylated *f*-SWCNT	788.7	0.197
Urazole *f*-SWCNT	820.8	0.154
ssDNA@urazole *f*-SWCNT	980.0	0.011

Dynamic Light Scattering Evidence. Although the hydrodynamic diameter increased upon ssDNA binding—consistent with the formation of a DNA corona or molecular entwinement—the PDI decreased by nearly an order of magnitude (from 0.154–0.197 to 0.011). These results indicate a narrow, monodisperse population of nanotube–DNA complexes rather than broad aggregation.

Nanotube-only and Pristine-SWCNT Control Experiments. As a control, pristine (unmodified) SWCNTs exhibited no intrinsic CD signal. When mixed with ssDNA, the pristine SWCNTs induced no appreciable perturbation of the ssDNA CD profile as shown in Figure S3. In contrast, the *f*-SWCNTs generated clear, base-dependent line-shape shifts, as described in the main text. These results confirm that the observed spectral responses originated from the grafted heterocycles rather than the graphitic backbone.

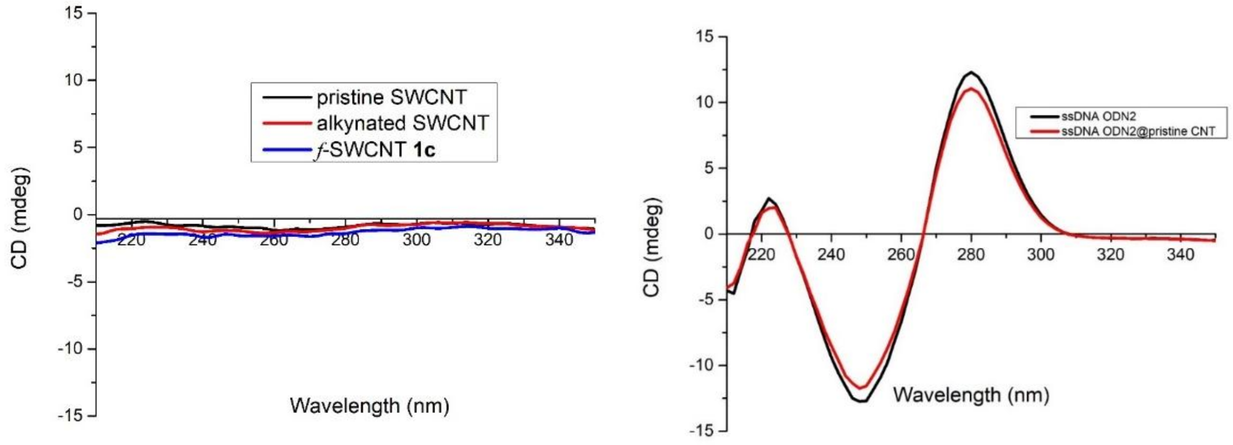

Figure S3. Circular dichroism (CD) spectra of control and functionalized SWCNT systems. Left: Pristine SWCNT, alkylated SWCNT, and *f*-SWCNT **1c**. Right: ssDNA (ODN2) alone and in the presence of pristine SWCNT.

*ssDNA ODN2 (Oiligonucleotide)- ssDNA 2 (5′-TCGAGTACGTCGCCGTCCAGCTCGA-3′).

In the present study, we performed CD measurements of the urazole ligand at multiple concentrations (e.g., 1.0 mg mL^{-1} and 10 mg mL^{-1}), as shown in Figure S4. The results exhibited systematic decreases in ellipticity and slight wavelength shifts with increasing concentration. These data offer a semi-quantitative correlation between ligand concentration and binding perturbation. Although full binding-constant determination was not performed, the observed CD variations consistently followed the ligand dosage and thus provided semi-quantitative evidence of interaction.[7–9]

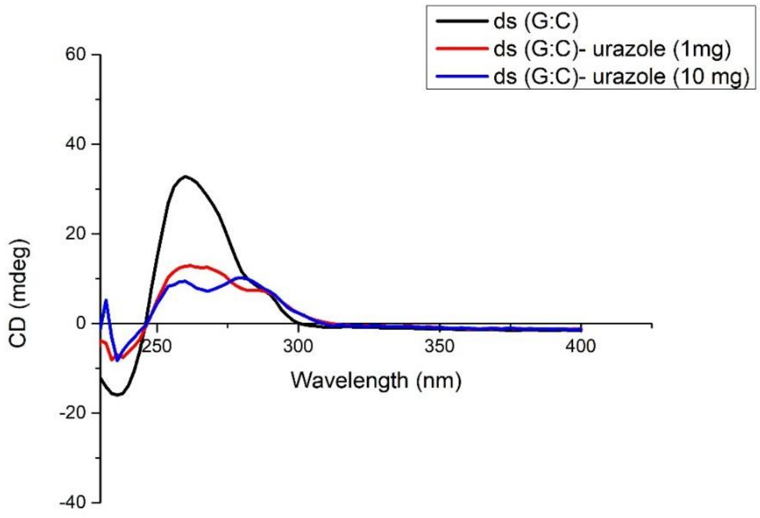

Figure S4. Circular dichroism spectra of ds(G·C) duplexes recorded in the absence and presence of urazole ligand at different concentrations.

References

1 Campidelli, S.; Ballesteros, B.; Filoramo, A.; Díaz Díaz, D.; de la Torre, G.; Torres, T.; Rahman, G. M. A.; Ehli, C.; Kiessling, D.; Werner, F.; Sgobba, V.; Guldi, D. M.; Cioffi, C.; Prato, M.; Bourgoin, J.-P. Facile Decoration of Functionalized Single-Wall Carbon

Nanotubes with Phthalocyanines via "Click Chemistry". *J. Am. Chem. Soc.* **2008**, *130*, 11503–11509.

2 Ziegler, K. J.; Gu, Z.; Peng, H.; Flor, E. L.; Hauge, R. H.; Smalley, R. E. Controlled Oxidative Cutting of Single-Walled Carbon Nanotubes. *J. Am. Chem. Soc.* **2005**, *127*, 1541–1547.

3 Weydemeyer, E. J.; Sawdon, A. J.; Peng, C.-A. Controlled Cutting and Hydroxyl Functionalization of Carbon Nanotubes through Autoclaving and Sonication in Hydrogen Peroxide. *Chem. Commun.* **2015**, *51*, 5939–5942.

4 Fagan, J. A.; Becker, M. L.; Chun, J.; Nie, P.; Bauer, B. J.; Simpson, J. R.; Hight-Walker, A. R.; Hobbie, E. K. Centrifugal Length Separation of Carbon Nanotubes. *Langmuir* **2008**, *24*, 13880–13889.

5 Hwu, J. R.; Landge, D. R.; Huang, W.-C.; Horng, J.-C.; Hu, Y.-C.; Hwang, K.-C.; Lin, C.-C.; Tsay, S.-C. Biochemical Nanotubes Containing Heterocycles as Artificial Strands for Pseudo Duplex and Triplex DNA Formation. *J. Phys. Chem. B* **2025**, *129*, 2903–2914.

6 Sandoval, S.; Kierkowicz, M.; Pach, E.; Ballesteros, B.; Tobias, G. Determination of the Length of Single-Walled Carbon Nanotubes by Scanning Electron Microscopy. *Methods X* **2018**, *5*, 1465–1472.

7 Garbett, N. C.; Ragazzon, P. A.; Chaires, J. B. Circular Dichroism to Determine Binding Mode and Affinity of Ligand–DNA Interactions. *Nat. Protoc.* **2007**, *2*, 3166–3172.

8 Greenfield, N. J. Using Circular Dichroism Spectra to Estimate Protein Secondary Structure. *Methods Enzymol.* **2004**, *383*, 282–317.

9 Longo, E.; Siligardi, G.; Hussain, R. Interaction of Blenoxane and Congeners Bleomycins A_2 and B_2 with Human Plasma Proteins Using Circular Dichroism Spectroscopy. *Int. J. Mol. Sci.* **2023**, *24*, 13598.
